# Host preference of field-derived *Schistosoma mansoni* is influenced by snail host compatibility and infection status

**DOI:** 10.1002/ecs2.4004

**Published:** 2022-04-08

**Authors:** Martina R. Laidemitt, Alyssa M. Gleichsner, Christopher D. Ingram, Steven D. Gay, Elizabeth M. Reinhart, Martin W. Mutuku, Polycup O Oraro, Dennis J. Minchella, Gerald M. Mkoji, Eric S. Loker, Michelle L. Steinauer

**Affiliations:** 1 Center for Evolutionary and Theoretical Immunology, Department of Biology, University of New Mexico, Albuquerque, New Mexico, USA; 2 Department of Biological Sciences, State University of New York, College at Plattsburgh, Plattsburgh, New York, USA; 3 College of Osteopathic Medicine of the Pacific Northwest, Western University of Health Sciences, Lebanon, Oregon, USA; 4 Department of Biological Sciences, Purdue University, West Lafayette, Indiana, USA; 5 Center for Biotechnology Research and Development, Kenya Medical Research Institute (KEMRI), Nairobi, Kenya

**Keywords:** *Biomphalaria*, choice chamber, field-derived, *Schistosoma mansoni*, schistosomiasis, western Kenya

## Abstract

Schistosome parasites cause a chronic inflammatory disease in humans, and recent studies have emphasized the importance of control programs for understanding the aquatic phases of schistosomiasis transmission. The host-seeking behavior of larval schistosomes (miracidia) for their snail intermediate hosts plays a critical role in parasite transmission. Using field-derived strains of Kenyan snails and parasites, we tested two main hypotheses: (1) Parasites prefer the most compatible host, and (2) parasites avoid hosts that are already infected. We tested preference to three *Biomphalaria* host snail taxa (*B. pfeifferi*, *B. sudanica*, and *B. choanomphala*), using allopatric and sympatric *Schistosoma mansoni* isolates and two different nonhost snail species that co-occur with *Biomphalaria*, *Bulinus globosus*, and *Physa acuta*. We also tested whether schistosomes avoid snail hosts that are already infected by another trematode species and whether competitive dominance played a role in their behavior. Preference was assessed using two-way choice chambers and by visually counting parasites that moved toward competing stimuli. In pairwise comparisons, we found that *S. mansoni* did not always prefer the more compatible snail taxon, but never favored an incompatible host over a compatible host. While parasites preferred *B*. *pfeifferi* to the nonhost species *B. globosus*, they did not significantly prefer *B. pfeifferi* versus *P*. *acuta*, an introduced species in Kenya. Finally, we demonstrated that parasites avoid infected snails if the resident parasite was competitively dominant (*Patagifer* sp.), and preferred snails infected with subordinates (xiphidiocercariae) to uninfected snails. These results provide evidence of “fine tuning” in the ability of schistosome miracidia to detect hosts; however, they did not always select hosts that would maximize fitness. Appreciating such discriminatory abilities could lead to a better understanding of how ecosystem host and parasite diversity influences disease transmission and could provide novel control mechanisms to improve human health.

## INTRODUCTION

Schistosomiasis is one of the great neglected tropical diseases of our time, caused by digenetic trematodes in the genus *Schistosoma*. Approximately 237 million people worldwide are infected with schistosomes, and the majority of infected individuals reside in sub-Saharan Africa (World Health Organization, 2020). Although progress has been made in limiting the spread of schistosomiasis through the use of mass drug administration campaigns, in March 2020, the World Health Organization reemphasized that snail host control will be critical in the elimination of schistosomiasis as a public health problem. Therefore, enhancing our understanding of the snail host-seeking behavior of field-derived schistosomes is of timely importance.

The snail-infective larval stages of schistosomes, miracidia, must navigate complex aquatic labyrinths, through a diverse community of freshwater snails, seeking out their compatible snail host species while avoiding similar snail species that are incompatible with infection, all within a life span of less than 24 h (Christensen, 1980; Esch et al., 2001; Rohr et al., 2015; Rynkiewicz et al., 2015). “Nonhost” gastropods could act as “decoy” hosts that exhaust or absorb miracidia, preventing the successful continuation of their life cycle (Civitello et al., 2015; Combes & Mone, 1987; Johnson et al., 2009; Keesing et al., 2010; Laracuente et al., 1979; Marszewska et al., 2020). Further, even when multiple snail species maintain infection in a transmission zone, the compatibility of those host species can be highly variable (Mutuku et al., 2021). Thus, selection is expected to fine-tune the parasites’ ability to discriminate between compatible and incompatible hosts (Johnson et al., 2019). Previous studies have shown that the recognition behaviors of a miracidium are largely mediated by snail chemical cues, such as glycoproteins, kairomones, or other peptides (Chernin, 1970; Fogarty et al., 2019; Haberl et al., 1995; Haberl & Haas, 1992; Theron et al., 1998; Wang et al., 2019). However, the degree of conservation of these chemical cues among and within host species is unknown.

In the Lake Victoria Basin, *Schistosoma mansoni* is transmitted by three different snail host taxa, *Biomphalaria pfeifferi*, *B. sudanica*, and *B. choanomphala*. Although the latter two taxa have been reported to be conspecific ecophenotypes (Standley et al., 2011; Zhang et al., 2018), we will refer to them by their original species designations for clarity. These three taxa exhibit distinct differences in habitat use: deep waters (occasionally found on the shoreline) of Lake Victoria for *B. choanomphala*, shallow shoreline of Lake Victoria for *B. sudanica*, and small rivers, dams, and impoundments surrounding Lake Victoria for *B. pfeifferi*. Each species also differs in its compatibility with *S. mansoni*. In the Lake Victoria Basin, their compatibilities range from highest compatibility (*B. pfeifferi*) to lowest compatibility (*B. sudanica*) (Laidemitt, Anderson, et al., 2019; Mutuku et al., 2014, 2017, 2019). Also, in this basin are several other species of snails that are incompatible with schistosome parasites, and thus are potential “decoys” if the parasites cannot differentiate between these species and compatible hosts (Johnson et al., 2009; Laidemitt, Anderson, et al., 2019).

Infection of snails by trematodes results in long associations (often lifelong), and the asexually proliferating progeny derived from a single miracidium come to occupy a large amount of the snail’s body for the production of cercariae (Gérard et al., 1993). Thus, multiple parasites infecting the same snail would be expected to compete for both space and nutrition. Although coinfections of trematode species and multiple genotypes of the same species occur (Eppert et al., 2002), experimental infections show that one trematode often outcompetes or preys upon the other, replacing it entirely (Fernandez & Esch, 1991; Hechinger et al., 2011; Lafferty et al., 1994; Laidemitt, Anderson, et al., 2019; Lim & Heyneman, 1972). Thus, if a snail is already infected with one or more trematodes, it would seem to be advantageous for a newly arriving miracidium (of any species) to avoid that infected snail because of the prospect of imminent competition for host space and resources, the possibility of consumption by preexisting predatory stages, or bystander immune responses triggered by the first infection (Allan et al., 2009; Sandland et al., 2007; Vannatta et al., 2020). For example, Allan et al. (2009) found that schistosome miracidia could distinguish between uninfected and already-infected hosts; likewise, Vannatta et al. (2020) found that *S. mansoni* will avoid *Biomphalaria glabrata* infected with the African echinostome, *Echinostoma caproni*, a competitively dominant parasite. There may also be situations where the infection of one trematode species might favor the subsequent infection of another species, where one trematode species may actually prefer a snail already infected with a different species (Southgate et al., 1989; Walker, 1979). Our previous work detailed the competitive interactions of the common trematode species infecting *Biomphalaria* in western Kenya (Carpenter et al., 2021; Laidemitt, Anderson, et al., 2019). In the new results we report here, we used four different species of parasites: *Schistosoma mansoni*, *Calicophoron sukari*, *Patagifer* sp., and a virgulate xiphidiocercariae species. Based on our previously published hierarchy, xiphidiocercariae are subordinate to *S. mansoni* (infection is taken over and replaced by *S. mansoni*), and *C. sukari* and *Patagifer* sp. are dominant to *S. mansoni*, with the echinostome, *Patagifer* sp., being the most dominant (Laidemitt, Anderson, et al., 2019).

We used choice chambers to test the extent to which field-derived Kenyan *S. mansoni* miracidia can differentiate between potential snail hosts. We predicted (1) that schistosome parasites will favor host species (compatible hosts) over nonhost (incompatible hosts) species, and they would choose uninfected snails over infected snails, and (2) that the strength of avoidance behaviors will increase when schistosomes encounter snails infected with competitively dominant parasites. Our unique study used snails recently derived from the field rather than laboratory lines that might be significantly impacted by either artificial selection or relaxed selection from the laboratory environment.

## MATERIALS AND METHODS

The behavioral assays were performed during two separate field seasons in Kenya during May 2017 and June 2019 with materials collected freshly for each set of trials. An effort was made to keep methodology as similar as possible between the two field seasons; however, minor differences are noted in the text below.

### Parasite and snail sources

**FIGURE 1:**
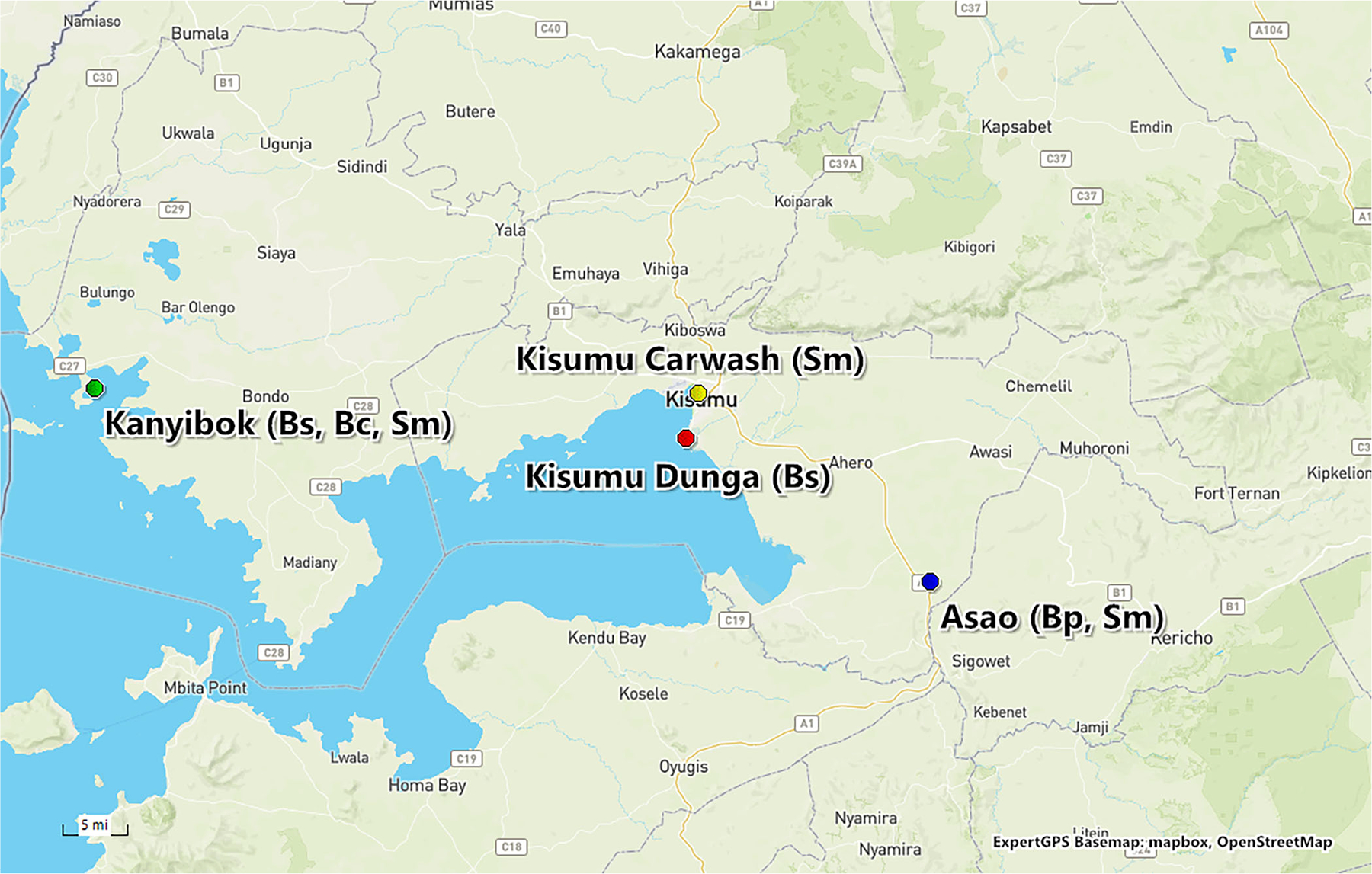
ExpertGPS BaseMap of locations in western Kenya in the Lake Victoria Basin where *Schistosoma mansoni* (Sm) parasites and *Biomphalaria* snails (*B. pfeifferi* [Bp], *B. sudanica* [Bs], *B. choanomphala* [Bc]) were collected. 1 mile = 1.609 km

*Schistosoma mansoni* eggs were collected and pooled from fecal samples from people living near schistosome transmission sites in the Lake Victoria Basin, Kenya. See the ethical statement below. Fecal samples were pooled from two to five people from various locations from lake or stream sites (Figure 1). Stool samples were processed as described by Mutuku et al. (2014) to concentrate eggs from the fecal samples. Briefly, the stool samples were pooled and homogenized in a blender and then filtered through a series of sieves in descending order (710, 425, 212, and 45 μm). The eggs are larger than the smallest sieve size (45 μm), so are captured on that sieve and then washed into a glass Erlenmeyer flask. Eggs were hatched in the flask using bottled water and ambient light. Miracidia that displayed positive phototaxis and normal shape and swimming behavior were used in the trials.

Three taxa of *Biomphalaria*, *B. pfeifferi* (Bp), *B. choanomphala* (Bc), and *B. sudanica* (Bs), and two species of nonhost snails, *Bulinus globosus* and *Physa acuta*, were used in the trials (Table 1). Abbreviations of the host taxa are used in the tables. Snails were collected from the field via long-handled scoops or dredging (Mutuku et al., 2019) and screened for parasitic infection by individually placing each snail into cell culture wells for 2 h under ambient light. Infected snails were separated from uninfected snails. Snail and cercaria identifications were based on taxonomic keys and previously published work (Brown, 1994; Frandsen & Christensen, 1984; Laidemitt et al., 2017; Laidemitt, Brant, et al., 2019; Schell, 1985). Subsequent molecular confirmation was performed. For the 2019 trials, some snail species could not be predictably collected from the field, so F1–F3 laboratory-bred snails were used instead. For example, in comparing *B. sudanica* with *B. choanomphala*, we used *B. sudanica* at the F1 generation and *B. choanomphala* at the F3 generation. Field-derived snails were always paired with field-derived snails, and laboratory-bred snails were always paired with other laboratory-bred snails. We also tested this in our choice chamber experiments to determine whether laboratory-bred or field-derived origins mattered. In some of the 2017 trials, we used F1s versus field-derived snails to determine whether there were differences between field-derived and F1s and we found that the parasites did not prefer one to the other (*F* = 9.39, *p* = 0.51). Snails used in each trial are detailed in Table 1. In the 2017 trials, because the field-derived snails may have had prepatent infections, snails were crushed after the trial was done, and if a snail was observed with a prepatent infection, the trial was redone.

Identities of a subsample of snail specimens were confirmed by sequencing a partial portion of the cytochrome oxidase 1 gene (*cox*1). Snail genomic DNA was extracted using the ENZA Mollusc Kit (Omega Bio-Tek, Norcross, CA). *Cox1* was amplified using the Folmer et al. (1994) primers LCO1490: 5′-GGT CAA CAA ATC ATA AAG ATA TTG G-3′ and HCO2198: 5′-TAA ACT TCA GGG TGA CCA AAA AAT CA-3′. The volume of each polymerase chain reaction (PCR) was 25 μL with 1 μL of 100 ng of DNA, 0.8 mM/L dNTPs, 2.5 mM/L MgCl_2_, 0.2 units of Ex Taq DNA (Clontech, Mountain View, CA), and 0.4 μM/L of each primer. PCR cycles were followed as described by Folmer et al. (1994), with the exception of an annealing temperature at 43.5°C. PCR fragments were separated by agarose gel electrophoresis and visualized with 0.5% GelRed nucleic acid gel stain and were purified using ExoSap-IT (Applied Biosystems, Foster City, CA). Both strands were sequenced using an Applied Biosystems 3130 automated sequencer and BigDye Terminator Cycle Sequencing Kit, version 3.1 (Applied Biosystems, Foster City, CA). DNA sequences were verified by aligning reads from the 5′ and 3′ directions using Sequencher 5.1 and manually corrected for ambiguous base calls (Gene Codes, Ann Arbor, MI). Not all snails could be sequenced due to permitting issues or amplification issues. Sequences generated in this study were submitted to GenBank accession number OL423116-OL423117. Snail and parasite sequences from the same or nearby locations have also been generated in other studies (Ebbs et al., 2018; Laidemitt et al., 2017; Laidemitt, Brant, et al., 2019; Zhang et al., 2018; C. Babbitt, 2021, personal communication).

### Choice chambers

The chamber used in this study is described in detail by Vannatta et al. (2020). The chamber was filled with 25 mL of bottled water. A barrier made with Play-Doh wrapped in plastic was used to divide the chamber into two equal halves. The snail(s) were placed on one or both sides of the divided chamber for 10 min while being observed by the experimenter to ensure the snail remained at the end of the divided chamber (away from the barrier). Once the 10 min was completed, the snail(s) and barrier were removed, ensuring that parasites could be counted after the trial. By eliminating signals that a parasite might gain from direct interaction with, or infection attempts of, a particular host individual, this approach also isolated the type of cue the parasite can use to make a choice to secretions from the host and its mucus. Once the snails were removed, 15 *S. mansoni* miracidia were placed via pipette in the center. After 10 min, the barrier was placed back in the center of the chamber, and the miracidia on each side of the divided chamber were counted by pipetting them out and into a petri dish. We waited for 10 min because in pre-control trials, less than 10 min was too short a time for the miracidia to choose, and longer than 20 min the miracidia would start to shed their plates and were more challenging to collect and count. Once a trial was completed, the chamber was cleaned with bleach and water before reuse. For the 2019 trials, choice chambers were set up with a small piece of lettuce on opposite sides, and with a forceps, snails were gently waved back in forth in the water for 3 s prior to releasing them in the chamber. These actions increased mucous secretions in the chamber, which presumably ensured the presence of chemical signals upon snail removal.

Controls for the choice chambers included no snail on either side of the divided chamber, as well as a snail versus no snail setup. The latter was performed for all three taxa of host snails (*Biomphalaria*), using two populations of miracidia, one sympatric and one allopatric. Details of the trials, including host species versus nonhost species, allopatric versus sympatric, low compatibility versus high compatibility, and infected versus uninfected snails, are given in Table 1.

### Data analysis

We used generalized linear mixed models (GLMMs) to determine whether *S. mansoni* miracidia choose snail hosts based on compatibility, sympatry, or infection status. GLMMs were used to ensure that the non-independence of miracidia within each trial (multiple miracidia per trial) was accounted for when determining whether miracidial choice was or was not random, given the choices presented. We used a binomial distribution in our GLMMs based on AIC (Akaike’s information criterion) comparisons. Within each trial, the number of miracidia that moved toward the predicted direction was compared with the number that moved in the opposite direction to determine whether a significant preference was shown toward one side. We used the package “glmmTMB” (Brooks et al., 2017) in the software R (version 3.6.1) for all data analyses. Preference indices (PI: number of miracidia in predicted direction – number of miracidia in opposite direction/total number of miracidia) were calculated for each treatment, treating each trial as an independent unit (Fischler et al., 2007). Positive values where the 95% confidence intervals do not overlap with zero indicate that the miracidia show a significant preference for the predicted choice (likewise, negative values that do not overlap with zero indicate significant preference in the opposite direction as predicted). Frequency distributions of PIs were examined to determine whether the behavior of miracidia appeared to be independent, or whether individuals tended to move in groups (the choice of others influences the choice of one). A normal distribution indicates independence, while a bimodal distribution suggests that other factors may influence choice. A Shapiro-Wilk test was used to assess whether PIs had a normal distribution (Royston, 1982). A bimodal distribution in the negative control treatment could potentially indicate group influences, while this distribution within a given treatment, or a positive control, may suggest that differences in snail stimuli (e.g., amount of mucus/signal) may be influencing behavior.

### Ethical statement

Human subjects were enrolled in our study to provide fecal samples as a source of *S. mansoni* to conduct the choice chamber experiments. Samples were collected and pooled from five primary school children from Obuon Primary School near Asao Stream, Kenya (−0.304915, 35.006476), in 2017 and 2019, from five adults from Kanyibok Village (−0.090124, 34.085722) in 2019, and from two adults from the Kisumu (Carwash) site (−0.095328, 34.750264) in 2017 that were discarded clinical (fecal) samples. Informed consent was obtained from all individual participants included in the study. The KEMRI Scientific and Ethics Review Unit (SSC No. 2373) and the UNM Institution Review Board (IRB 821021–1) approved all aspects of this project involving human subjects. Individuals who were tested and found positive for *S. mansoni* were treated with praziquantel following standard protocols. Details of recruitment and participation of human subjects for fecal collection are described in Mutuku et al. (2014).

## RESULTS

### Do parasites orient toward snail cues?

When no snails were introduced to the chamber (Table 2), miracidia showed no apparent preference for either side of the divided chamber (*Z* = −0.40, *p* = 0.629). When given a choice between a host snail and an empty side of the chamber, the parasites were significantly more likely to choose the side with a snail for each of three compatible host taxa (*B. pfeifferi*: average *Z* = 6.42, *p* < 0.001; *B. sudanica*: average *Z* = 4.37, *p* < 0.001; *B. choanomphala*: average *Z* = 5.34, *p* < 0.001). Preference indices confirmed these results and were normally distributed, indicating independence of miracidial choice. Parasites always chose the snail in control trials where the choice was no snail versus snail, and the strength of that choice did not significantly differ whether the snail was a laboratory-reared or field-derived snail. We tested this in our choice chamber experiments to determine whether laboratory-bred or field-derived origins mattered. In some of the 2017 trials, we used F1s versus field-derived snails to determine whether there were differences between field-derived and F1s and we found that the parasites did not prefer one to the other (*F* = 9.39, *p* = 0.51). Likewise, the field or laboratory status of the snail did not impact choice in our *B. sudanica* versus *B. pfeifferi* trials.

### Do parasites choose the most compatible host?

For these trials, we first compared compatible snail species versus incompatible snail species, and then, we compared compatible snail species that exhibited different levels of compatibility (Table 2). When given a choice between *B. pfeifferi* (host - compatible) and *B. globosus* (nonhost - incompatible), *S. mansoni* miracidia were found on *B. pfeifferi*’s side of the divided chamber more often (*Z* = −2.01, *p* = 0.045). There was no significant difference in choice between *B. pfeifferi* (host) and *P. acuta* (nonhost - incompatible) (*Z* = 1.48, *p* = 0.139). Preference indices confirmed these findings and independence of miracidia choice.

As shown in Table 2, when given a choice between *B. sudanica* (less compatible) and *B. choanomphala* (more compatible), parasites chose the more compatible host (*Z* = 2.29, *p* = 0.022). However, the choice between *B. sudanica* (less compatible) and *B. pfeifferi* (more compatible) did not differ significantly overall (*Z* = −5.73, *p* = 0.566). Two sets of trials with this snail combination were completed, each set using sympatric and allopatric snail hosts from different locations. When pooled, the results are not significant, as stated above. However, examined separately, we see location-specific effects. In trials using a full factorial design in which Asao and Kanyibok miracidia (see Figure 1) were given a choice between Asao-collected *B. pfeifferi* and Kanyibok-collected *B. sudanica*, we found that Asao miracidia significantly selected the sympatric host (*Z* = −2.278, *p* = 0.023), while Kanyibok miracidia exhibited no clear choice (*Z* = 0.454, *p* = 0.649) (Appendix S1: Table S1). In the second set of trials, miracidia from Kisumu and Asao were given a choice between *B. pfeifferi* (from Asao) and *B. sudanica* (from Kisumu). In these trials, there were no significant choices made by either Asao miracidia (*Z* = 1.01, *p* = 0.311) or Kisumu miracidia (*Z* = −0.50, *p* = 0.617) (Appendix S1: Table S2). Allopatric and sympatric results are in Appendix S1: Tables S1 and S2.

### Do parasites avoid infected hosts?

As shown in Table 3, when given a choice between a snail patently infected with *S. mansoni* or an uninfected snail, parasites showed no significant preference (*Z* = −1.62, *p* = 0.106). This was contrary to our hypothesis that the parasites would avoid a snail already infected with conspecifics. All infected snails were patent, “shedding” cercariae.

We also hypothesized that infected/uninfected host preference would be stronger when the snail was infected with a competitively dominant parasite (one that outcompetes *S. mansoni* and dominates the infection). We tested representatives from two groups of parasites that are dominant to *S. mansoni*, an echinostome, *Patagifer* sp., and an amphistome, *C. sukari* (Table 3). Parasites chose the noninfected snail more often than the infected snail when the snail was infected with *Patagifer* sp. (*Z* = 2.85, *p* = 0.004). Interestingly, when the snail was infected with a nonreplicative ontogenetic stage of *Patagifer* sp. (metacercariae), there was no significant difference in preference between the uninfected and infected snail (*Z* = 1.06, *p* = 0.291). Also, with the other “dominant” parasite, *C. sukari*, there was no significant preference between the infected and uninfected snail (*Z* = −1.29, *p* = 0.198).

Finally, when given a choice between the subordinate xiphidiocercariae-infected snail and an uninfected snail (Table 3), the parasite chose the *infected* snail more often (*Z* = −2.06, *p* = 0.039). Preference indices confirmed these findings and indicated that miracidia made choices independently.

## DISCUSSION

After hatching from the egg, a schistosome parasite has a short time (<24 h) to locate and invade a compatible snail species (Maldonado et al., 1949; Prah & James, 1977). They must navigate complex environments with diverse communities of snails, many of which are already infected by an equally diverse array of trematode parasites. Our study supports previous findings that miracidia actively navigate toward snail cues, but it goes further to determine the extent to which field-derived miracidia can differentiate these snail-derived cues. With a series of binary choices, we asked whether parasites can select hosts that will increase their fitness. Our study is among the few studies that utilize African snails and schistosomes either directly derived or recently derived from the field in choice chamber experiments (see Allan et al., 2009). One of the limitations of this study was the testing system that allowed only binary choices and that not every combination of possible choices could be tested in the time that we had access to field material.

When given a choice between highly compatible host species (*B. pfeifferi*) versus incompatible nonhost species (*Bulinus globosus* or *P. acuta*), the parasites significantly oriented toward the compatible host when *B. globosus* was the alternative, but not when *P. acuta* was the alternative. In the latter case, the parasites did not show a significant preference for either snail. Previous choice experiments with *S. mansoni* and various snails show mixed results with regard to preference between compatible host and incompatible nonhost species (Basch, 1976; Chernin, 1970; Hassan et al., 2003; Kalbe et al., 1996). However, the pattern in previous studies appears to be that the ability to discern between compatible hosts and incompatible nonhosts is stronger with more distantly related snails, perhaps due to the conservation of biochemical signals (Allan et al., 2009; Christensen, 1980; Kalbe et al., 1996). Our results show the opposite. *Bulinus* and *Biomphalaria* belong to the family Planorbidae, while *Physa* belongs to a different family, the Physidae. One possible explanation for this result is that *P. acuta* is a relatively recent invader in Africa, sometime after the 1870s (Ebbs et al., 2018). Thus, the ability for *S. mansoni* miracidia to discern between *Bulinus* and *Biomphalaria* species may have evolved from the long history of co-occurrence (>1 million years ago) (DeJong et al., 2001; Neubauer et al., 2017). It has also been noted in other studies that avian schistosomes did not choose compatible host species versus incompatible alien species when given a choice in snail-conditioned choice chamber experiments (Marszewska et al., 2020). More comparisons of other planorbids and alien species against compatible host species are needed to test this hypothesis fully. It should also be noted that although the chances are low, penetrating a nontarget intermediate host is not necessarily always a dead end. It might lead to a host-switching event that may favor the parasite’s transmission in the future by broadening host or geographic range, as was the case for other schistosome species (Brant & Loker, 2013; Combes & Mone, 1987). Also, our results exemplify the possibility that an introduced species can have more subtle effects, such as diluting the impact of human parasites, than normally considered.

Within the Lake Victoria Basin, *S. mansoni* can infect three taxa of *Biomphalaria* with differing levels of success. We hypothesized that given a choice between two compatible host species, the parasite would prefer the most compatible. Results from *B. choanomphala* (more compatible) versus *B. sudanica* (less compatible) supported this hypothesis. This finding is interesting, because *B. choanomphala* is genetically quite similar to *B. sudanica* (Standley et al., 2014; Zhang et al., 2018), but shows clear differences in habitat use (Gouvras et al., 2017; Mutuku et al., 2019) and is more compatible with Kenyan *S. mansoni* (Mutuku et al., 2021). Our data suggest that schistosomes can distinguish between these snails (even if researchers cannot and bin them into the same species) and orient toward *B. choanomphala* when given a choice.

When the choice was *B. pfeifferi* (highly compatible) versus *B. sudanica* (least compatible), there was no significant preference between these species. These two species of snails are *rarely* found in sympatry, with *B. pfeifferi* in streams and small impoundments and *B. sudanica* in papyrus swamps associated with larger water bodies (Brown, 1994). Nested within this trial, we also tested whether the source of the parasite mattered. We predicted that parasites would show a greater preference for sympatric hosts due to local adaptation. Our trials used parasites sympatric to either the *B. pfeifferi* snail or the *B. sudanica* snail (parasites collected from humans who inhabit the same general locality). Due to logistics, the *B. sudanica* snail source and corresponding parasite source differed between field seasons (either Kanyibok or Kisumu), but the *B. pfeifferi* source and corresponding parasite source remained the same (Asao). Parasites from Asao chose *B. pfeifferi* (sympatric) over *B. sudanica* from Kanyibok (allopatric), but Kanyibok parasites showed no significant preference to either snail species. In contrast to these results, when the trials compared *B. sudanica* and *B. pfeifferi* from Kisumu and Asao, no significant preferences were found, and parasite source was not a significant variable in the model. It is possible that there were host genotypic differences that play a role and that the paired choice matters (e.g., Asao snail vs. Kisumu snail or Asao snail vs. Kanyibok snail).

Our results are similar to previous tests of different schistosome and snail systems, which show mixed results. Allan et al. (2009) assessed *S. haematobium* choice between two different compatible species of *Bulinus*, and the miracidia did not show significant preference. Kalbe et al. (1996) found that an Egyptian strain of *S. mansoni* miracidia preferred snail-conditioned water from *Biomphalaria alexandrina* (sympatric) to *B. glabrata* (allopatric); however, miracidia from two Brazilian strains did not show a preference between snail-conditioned water from the two snail species.

In trials comparing preference to infected or uninfected snails, *S. mansoni* miracidia avoided snails infected with the most dominant trematode in the hierarchy, *Patagifer* sp., but showed no preference when the infection was with either *C. sukari* (competitively dominant to *S. mansoni*, but subordinate to *Patagifer* sp.) or *S. mansoni* (conspecific), and *favored* snails infected with xiphidiocercariae (competitively subordinate). These results are generally in line with our expectations from the dominance hierarchy, but there are important differences. Because *C. sukari* is dominant to *S. mansoni*, we predicted that *S. mansoni* would avoid snails infected with this species. One potential explanation is the complicated interdependence of *C. sukari* on *S. mansoni*. Although *C. sukari* can invade and establish within *B. pfeifferi*, it cannot complete its development unless the snail becomes infected with *S. mansoni* (Laidemitt, Anderson, et al., 2019); thus, there could be selection pressure on *C. sukari* to hide signals of snail infection.

Vannatta et al. (2020) also found a preference for uninfected *B. glabrata* versus *B. glabrata* infected with *Echinostoma caproni*, also a competitively dominant echinostome parasite to *S. mansoni*. Echinostomes are known to engage in direct (predatory) and indirect antagonism against schistosomes (Hechinger et al., 2011; Lim & Heyneman, 1972; Sandland et al., 2007). These data suggest that either echinostome ESPs (excretory–secretory products) are being detected in the snail-conditioned water, or snail ESPs are being modified from the infection and then being detected in the snail-conditioned water that may act as miracidial deterrents. For example, it has been shown in the *B. glabrata*–*S. mansoni* system that a peptide, called P12, is secreted from *B. glabrata*, to which *S. mansoni* is significantly attracted (Fogarty et al., 2019), and there are likely other peptides or metabolites that are being excreted or secreted that *S. mansoni* miracidia actively avoid. Further liquid chromatography–mass spectrometry studies are needed to characterize ESPs released by the larval stages of echinostomes within their snail. *Patagifer* species are of interest because they commonly infect *Bulinus* and *Biomphalaria*, both of which transmit human schistosomes (Laidemitt, Brant, et al., 2019). One possibility for future testing is that the echinostome-infected snail might release less of a particular signal simply as a by-product of the infection by reduction in mucous secretions.

In regard to *Patagifer* sp. (echinostome) avoidance, there was also a trend (although not significant) that *S. mansoni* miracidia preferred uninfected snails to those infected with *Patagifer* sp. metacercariae. Metacercariae are encysted stages that are nonpredatory and nonreplicative within the snail host. They also tend to be found in tissues of the snail different from those occupied by sporocysts, rediae, or cercariae, and metacercariae are typically common in wild snail populations. We found it difficult to find field snails that were not infected with metacercariae of one species or another.

An interesting result was that *S. mansoni* miracidia preferred *B. pfeifferi* infected with virgulate xiphidiocercariae to uninfected *B. pfeifferi*. It is possible the sporocysts of xiphidiocercariae may suppress or interfere with the anti-trematode immune system of the snail, improving the chances that *S. mansoni* can establish an infection (Adema & Loker, 2015; Hanington et al., 2012; Lie, 1982). Alternatively, schistosome parasites may derive nutrients from the xiphidiocercariae within the snails, and these extra nutrients might boost its fitness. Similarly, Vannatta et al. (2020) found that *E. caproni* miracidia significantly chooses *S. mansoni* (subordinate)-infected *B. glabrata* over uninfected *B. glabrata* for likely similar reasons.

## SUMMARY AND CONCLUSIONS

Our findings support previous results that miracidia are attracted to cues produced by snails and/or from the parasites within snails. Further, miracidia sensory receptors may be able to differentiate among compatible host species and avoid coinfection within a snail if the resident parasite is competitively dominant. However, our results demonstrate that predictions concerning host preference are not necessarily generalizable, indicating complexity in the system. Further work is needed to identify the signals involved in sensing hosts and how these signals vary within field-derived snail populations and between snail species. The advent of dramatically improved detection technology coupled with an increasing amount of information about snail and parasite genomes will enable further elucidation of the chemical language of trematodes, including within their snail hosts. This is especially important when the snails involved transmit parasites that cause infections in millions of people. We emphasize the importance of investigating this chemical language as closely as possible as it is revealed under conditions of natural transmission.

## Supplementary Material

Appendix s1

## Figures and Tables

**TABLE 1:** Snail source table of their collection location, habitat type, compatibility with *Schistosoma mansoni*, and whether they were laboratory- or field-derived

Snail species	Location collected	Habitat	Compatibility with *S. mansoni*	Laboratory-reared or field-derived
*Biomphalaria pfeifferi*	Asao −0.316062, 35.006040	Stream	High	Field-derived
*Bulinus globosus*	Asao −0.316062, 35.006040	Stream	None	Field-derived
*Physa acuta*	Asao −0.316062, 35.006040	Stream	None	Field-derived
*Biomphalaria choanomphala*	Kanyibok Village −0.091337, 34.085053	Deepwater Lake Victoria	Medium-high	Laboratory (three generations of breeding in the laboratory)
*Biomphalaria sudanica*	Kanyibok Village −0.089581, 34.085928	Shoreline Lake Victoria	Low	Laboratory (one generation of breeding in the laboratory)
*Biomphalaria sudanica*	Dunga (Kisumu) Village −0.144784, 34.736261	Shoreline Lake Victoria	Low	Field-derived

**TABLE 2:** Statistical results by treatment indicating whether parasites can differentiate between different host taxa

Treatment	Estimate	SE	*Z*	*N*	*p*	Direction	Percentage toward predicted
No snail versus no snail	−0.04	0.16	−0.24	11	0.807	None	48.5
Bc versus no snail	1.06	0.20	5.34	28	**<0.001**	**Toward snail**	**73.34**
Bs versus no snail	0.83	0.19	4.37	36	**<0.001**	**Toward snail**	**68.94**
Bp versus no snail	1.18	0.18	6.42	53	**<0.001**	**Toward snail**	**75.54**
Bs versus Bc	0.47	0.21	2.29	19	**0.022**	**Toward Bc**	**60.54**
Bs versus Bp	−0.10	0.18	−0.57	51	0.566	None	53.78
Bp versus *Physa acuta*	0.33	0.22	1.48	12	0.139	None	57.24
Bp versus *Bulinus globosus*	−0.49	0.25	−2.01	9	**0.045**	**Toward Bp**	**63.97**

*Note*: The significance, direction of the choice, and average % of miracidia (averaged across all trials in a treatment) are in bold if past a significance threshold of *p* < 0.05. *Biomphalaria* sp. host names are abbreviated, with Bc representing *Biomphalaria choanomphala*, Bs representing *B. sudanica*, and Bp representing *B. pfeifferi*.

**TABLE 3:** Statistical results by treatment indicating whether parasites can differentiate between hosts of varying infection status

Treatment	Estimate	SE	*Z*	*p*	*N*	Direction	Percentage toward predicted
No snail versus no snail	−0.06	0.16	−0.40	0.692	11	None	48.5
Laboratory F1 Bp uninfected versus no snail	0.80	0.23	3.48	**<0.001**	12	**Toward snail**	**66.5**
Field-uninfected Bp versus no snail	0.91	0.23	3.94	**<0.001**	12	**Toward snail**	**70.2**
*Schistosoma mansoni*-infected Bp versus uninfected Bp	−0.37	0.23	−1.62	0.106	11	None	39.3
*S. mansoni*-infected Bp and Bs versus *Patagifer* sp.-infected Bp and Bs	0.24	0.24	0.99	0.321	9	None	54.2
Uninfected Bp versus Bp-infected with *Patagifer* sp.	0.53	0.19	2.85	**0.004**	34	**Toward uninfected**	**61.1**
Uninfected Bp versus Bp-infected with *Patagifer* sp. Metacercariae	0.24	0.22	1.06	0.291	12	None	54.8
Uninfected Bp versus Bp-infected with *C. sukari*	−0.29	0.23	−1.29	0.198	11	None	58.9
Uninfected Bp versus Bp-infected with Xiphidiocercariae	−0.48	0.23	−2.06	**0.039**	11	**Toward infected**	**36.4**

*Note*: The significance, direction of the choice, and average % of miracidia (averaged across all trials in a treatment) are in bold if past a significance threshold of *p* < 0.05. *Biomphalaria* sp. host names are abbreviated, with Bc representing *Biomphalaria choanomphala*, Bs representing *B. sudanica*, and Bp representing *B. pfeifferi*.

## Data Availability

Data (Gleichsner, 2022) are available from Zenodo: https://doi.org/10.5281/zenodo.6028760.
