# Supplementary material for: Host preference of field-derived Schistosoma mansoni is influenced by snail host compatibility and infection status
Source: Ecosphere. Author manuscript; Available in PMC 2022 Oct 24. (PMC9592064; doi:10.1002/ecs2.4004)
Supplement: Appendix s1 [file NIHMS1841778-supplement-Appendix_s1.pdf]

Ecosphere

Host preference of field-derived *Schistosoma mansoni* is influenced by snail host compatibility and infection status

Martina R. Laidemitt, Alyssa M. Gleichsner, Christopher D. Ingram, Steven D. Gay, Elizabeth M. Reinhart, Martin W. Mutuku, Polycup Oraro, Dennis J. Minchella, Gerald M. Mkoji, Eric S. Loker, and Michelle L. Steinauer

Appendix S1

Table S1. Allopatric vs. sympatric 2019 data. Bp vs. Bs

**Treatment**	**Estimate**	**Std. Error**	**Z value**	**P value**	**Result**
Intercept	-0.039	0.163	-0.244	0.807	
Asao Miracidia	-0.488	0.214	-2.278	**0.023**	**Asao miracidia significantly choose Bp snails**
Kanyibok Miracidia	0.115	0.253	0.454	0.649	NS

Table S2. Allopatric vs. sympatric 2017 data. Bp vs. Bs.

**Treatment**	**Estimate**	**Std. Error**	**Z value**	**P value**	**Result**
Intercept	-0.039	0.163	-0.244	0.807	
Asao Miracidia	0.222	0.219	1.013	0.311	No difference
Kisumu (Carwash) Miracidia	-0.109	0.219	-0.500	0.617	No difference
